# Quantifying Bite Forces for Solid Foods: Implications for Patients Postmandibular Reconstruction

**DOI:** 10.1002/hed.70031

**Published:** 2025-09-03

**Authors:** Jacob J. Clark, Emma K. Charters, Kai Cheng, Boyang Wan, Masako Dunn, Tim G. H. Manzie, Dale G. Howes, Elizabeth C. Ward, Qing Li, Jonathan R. Clark

**Affiliations:** ^1^ School of Biomedical Engineering, Faculty of Engineering, The University of Sydney Darlington Australia; ^2^ Department of Head and Neck Surgery Chris O'Brien Lifehouse Camperdown Australia; ^3^ Royal Prince Alfred Institute of Academic Surgery, Sydney Local Health District Camperdown Australia; ^4^ School of Aerospace, Mechanical and Mechatronic Engineering, Faculty of Engineering, The University of Sydney Camperdown Australia; ^5^ NHMRC Centre of Research Excellence for Applied Innovations in Oral Cancer, The University of Sydney Sydney Australia; ^6^ Central Clinical School, Faculty of Medicine and Health, The University of Sydney Camperdown Australia; ^7^ School of Dentistry, Faculty of Medicine and Health, The University of Sydney Camperdown Australia; ^8^ Centre for Functioning and Health Research, Metro South Health, Queensland Health Brisbane Australia; ^9^ School of Health and Rehabilitation Sciences, Faculty of Health, Medicine and Behavioral Sciences, The University of Queensland St Lucia Australia

**Keywords:** bite force, dietary recommendations, finite element analysis, IDDSI, mandibular reconstruction

## Abstract

**Background:**

Bite forces required to masticate different food consistencies remain unknown, complicating dietary guidelines following mandibular reconstruction. This study quantifies the forces required for solid foods and estimates safe bite limits postreconstruction.

**Methods:**

Twenty food items were prepared according to IDDSI Levels 5 through 7b. Incisor and molar bite forces were measured using a custom‐built force meter. Finite element (FE) analysis simulated safe force thresholds for single‐, double‐, and triple‐segment mandibular reconstructions.

**Results:**

Bite force increased with food consistency, ranging from 4.6 N (IDDSI 5, incisors) to 45.4 N (IDDSI 7b, molars). Molars required higher forces than incisors (mean difference 9.0 N, *p* < 0.001). FE analysis indicated safe bite forces of30–115 N depending on reconstruction complexity and bone union. IDDSI 5 foods were safe in all cases, while higher levels often exceeded thresholds.

**Conclusion:**

These findings support evidence‐based dietary recommendations after mandibular reconstruction using the IDDSI framework.

## 1 Introduction

Osseous free flap reconstruction of the mandible aims to restore bony continuity, facial esthetics, and oral function [1, 2]. In this context, mastication depends on the mechanical integrity of the reconstructed mandibular system, without which, excessive loading risks hardware failure due to plate deformation or fracture and screw loosening due to osteolysis [3]. Healthy adult bite forces range from 108 to 228 N for incisors and 216–544 N for molars [4, 5], but currently, the safe bite force limits for patients recovering from mandibular reconstruction, especially preceding bone union, remain unknown. A puree (no chew') diet may be prescribed for 6–8 weeks after mandibular reconstruction to mitigate the risk of hardware failure or nonunion. However, this reduces the enjoyment of food, inhibits early oral rehabilitation, and may impair nutrition. Furthermore, early mechanical loading can encourage bone regeneration and osseointegration [6].

Mastication of food is an intuitive way of providing early loading to the jaw, assuming that the masticatory forces can be reliably quantified and applied within safe levels. To date, this remains untested in the setting of mandibular reconstruction because the safe loading limits and the bite forces required for different food textures are unknown. Finite element (FE) analysis can be used to predict the biomechanical behavior of the reconstructed mandible and estimate safe loading limits based on computed tomography (CT) data [7]. Factors such as texture, density, liquid content, and the tooth's surface area and topology influence the forces involved in mastication. The International Dysphagia Diet Standardization Initiative (IDDSI) categorizes the texture of foods as shown in Table 1 [8]. One of the testing methods is the “fork pressure test,” which uses a fork to compress and deform a 1.5 cm [3] food item with light pressure (until the thumb blanches). This is a requirement for food prepared for IDDSI Levels 5, 6, and 7 meals. The Iowa Oral Performance Instrument (IOPI) was used to quantify the pressure at which the thumb blanches (17 kPa) [9], however, it is unknown whether the fork pressure test correlates with masticatory forces, as there is a little understanding of the forces required to masticate the foods in each of the IDDSI food categories. Consequently, it is unclear which if any of these solid food consistencies are safe to consume following mandibular reconstruction. The aim of this study was to first estimate the force required to bite different types of solid foods in each IDDSI category using a replicate model of incisor and molar teeth, and then to combine this data with FE simulations of different mandibular reconstructions to determine what food consistency is likely to be safe immediately following surgery.

**TABLE 1:** Description of IDDSI Levels 5–7b.

Level	Description
5—minced and moist	Very soft, small moist lumps, minimal chewing ability needed
6—soft and bite‐sized	Soft and bite‐sized, tender and moist throughout, with no thin liquid leaking or dripping from the food. Chewing ability needed.
7a—easy to chew	Normal or everyday foods of soft/tender textures only, that are developmentally and age appropriate. Requires biting and chewing ability.
7b—regular	Normal everyday foods of various textures that are developmentally and age appropriate. Biting and chewing ability needed.

Abbreviation: IDDSI = International Dysphagia Diet Standardization Initiative.

## 2 Materials and Methods

### 2.1 Device Assembly

**FIGURE 1:**
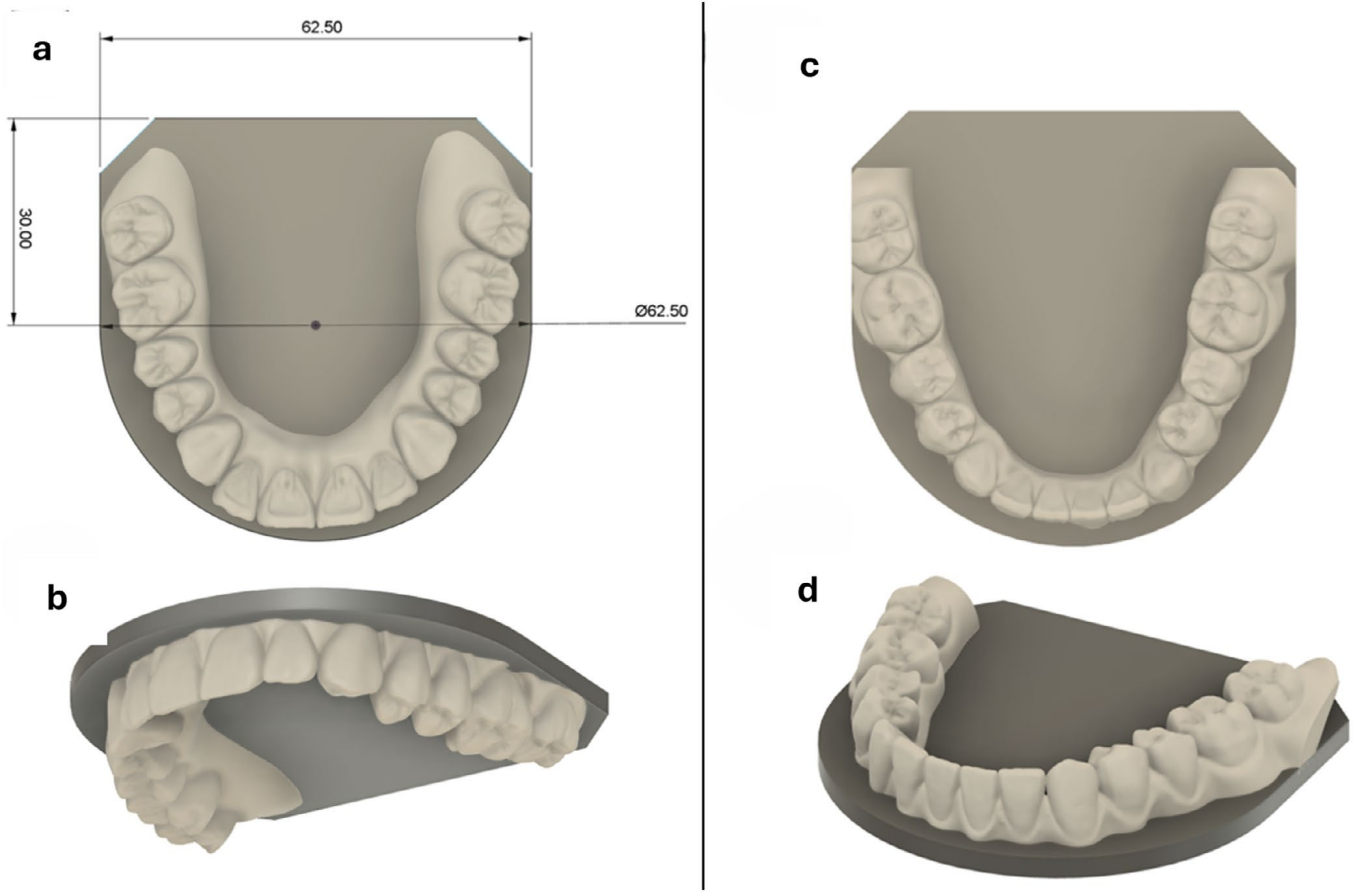
3D virtual model of dental prosthesis with attached platform. Inferior (a) and perspective (b) view of maxillary teeth. Superior (c) and perspective (d) view of mandibular teeth. [Color figure can be viewed at wileyonlinelibrary.com]

**FIGURE 2:**
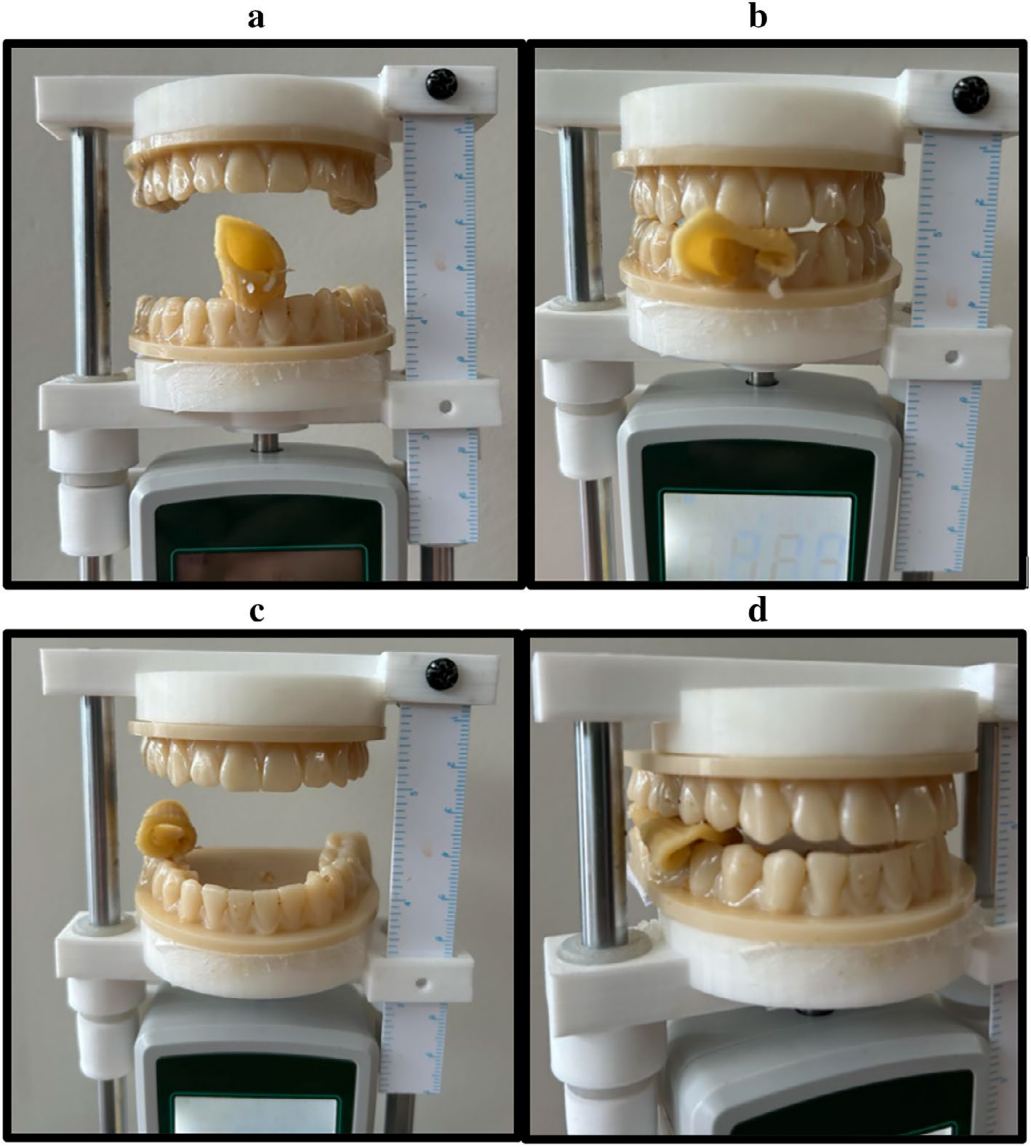
Measurement of occlusal load using interincisal distance and force meter (IIDFM). Demonstrates occlusal load is shown at the incisors (a, b) and molars (c, d) during mastication of cooked pasta. [Color figure can be viewed at wileyonlinelibrary.com]

A 3D model of maxillary and mandibular teeth was generated from CT data taken from a healthy adult male and fabricated in Crowntec resin (Saremco, Rebstein, Switzerland) using a Pro 4 K Printer (Asiga, Sydney, Australia) as shown in Figure 1. The dental prosthesis was secured to a purpose‐built interincisal distance and force meter (IIDFM) used to quantify occlusal load as described by Charters et al. [10]. The IIDFM device maintained a normal dental occlusion to simulate bite mechanics and to obtain valid measurements (Figure 2).

### 2.2 Food Sampled

A total of 79 foods were analyzed from IDDSI Level 5 (*minced and moist)*, 6 *(soft and bite‐sized)*, 7a *(easy‐to‐chew*), and 7b *(regular)* (Table 1) [8, 11]. Within each level, at least two meats, two fruits or vegetables, and two carbohydrates were sampled. Food preparation followed IDDSI guidelines, including standardized size (1.5 × 1.5 cm), and by conducting the fork pressure, spoon pressure, and drip tests to ensure validity [11].

### 2.3 Force Assessment

The modified IIDFM device records the peak force in Newtons (N) required to compress foods between the incisor and molar teeth on the model until fully penetrated. Each food item was tested six times (three incisor and three molar), yielding 480 force measurements. A 10% interrater reliability test was employed with two raters (J.R.C. and E.C.W.). The second rater resampled two foods from each category to evaluate the consistency of the testing methodology.

### 2.4 FE Analysis

**FIGURE 3:**
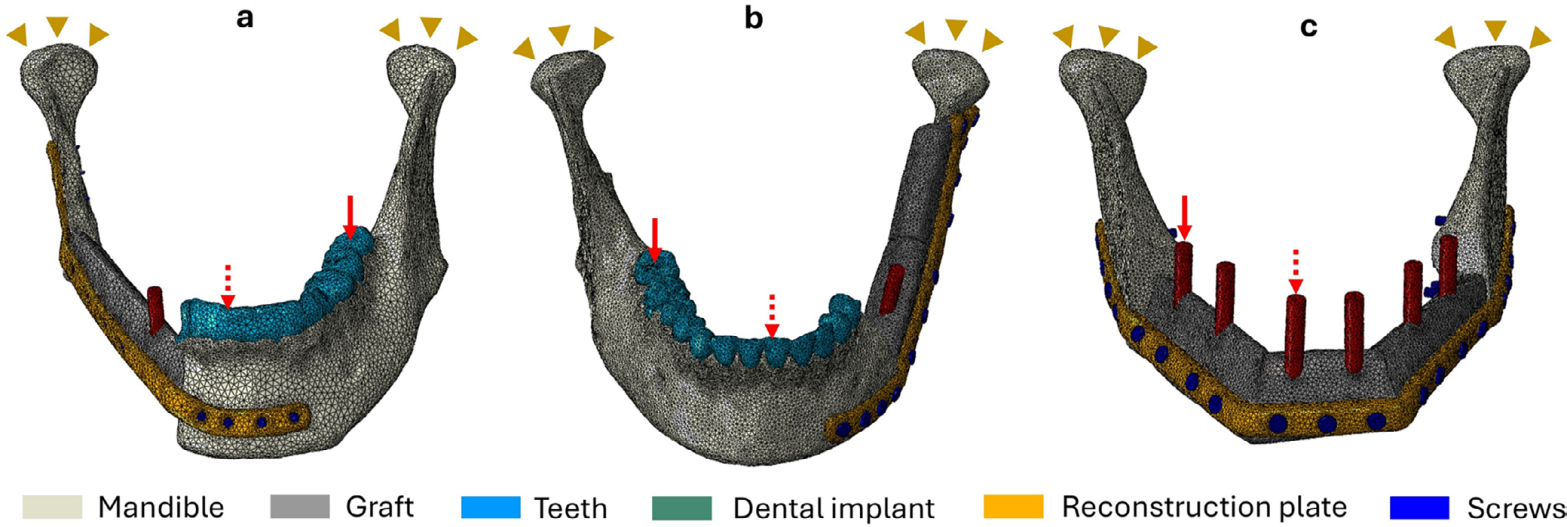
Patient‐specific FE models of mandibular reconstruction from CT data for (a) single‐segment, (b) double‐segment, and (c) triple‐segment fibula free flap mandibular reconstructions. Yellow triangles represent temporomandibular joint (TMJ) restraints. Solid red arrows indicate posterior (molar) loading; dotted red arrows indicate anterior (incisor) loading. [Color figure can be viewed at wileyonlinelibrary.com]

FE analysis was conducted using ABAQUS (ABAQUS Inc., Providence, RI) to simulate the biomechanical behavior of the differently reconstructed mandibles under varying occlusal loads. A four‐node tetrahedral element (C3D4) was used to mesh the prostheses and bones. A mesh convergence study was performed to balance computational accuracy and efficiency, resulting in models consisting of approximately 187 768 elements and 113 898 degrees of freedom on average. The von Mises stress in the reconstruction plate was calculated to evaluate the safety of various configurations, including single, double, and triple‐segment fibula free flap reconstructions of the mandible under incisor and molar loading. In all the representative mandibular FE models, the temporomandibular joints (TMJs) were fully constrained as illustrated in Figure 3 to facilitate comparison [12, 13]. Two contact conditions were adopted at the bone‐graft interface: the first scenario assumed a lack of bone union, while the second scenario mimicked the presence of bone union between the graft interfaces. Therefore, the interface contact conditions differed between the two scenarios. The first (nonunion) scenario assumed a frictional contact coefficient of 0.1 to mimic the immediate postoperative period. Conversely, in the second (union) scenario, a tie constraint was used at the graft interface [14]. Additionally, a frictional grip connection with a coefficient of 0.3 was applied at the bone‐plate interface when contact occurred [15].

All materials involved were assumed to be isotropic and linear elastic, and the required material properties, Young modulus, *E* (MPa) and Poisson ratio *ν* were taken from the existing literature and were given as follows: for bone, 9200 and 0.33, for teeth, 19 800 and 0.3, and for titanium plates and screws, 110 000 and 0.33, respectively [16, 17]. The peak stresses were compared to the yield strength of Grade 2 titanium, which ranges from 275 to 410 MPa, with the lower bound of 275 MPa adopted to ensure conservative estimates of safety. After this, FE simulations were performed to inversely estimate safe bite force limits in various mandibular configurations, according to the strength of the reconstruction plate [18, 19].

### 2.5 Statistical Analysis

Data was analyzed in R (v4.4.2) on RGui [20] using the tidyr package [21]. A *t* test compared bite forces across different IDDSI categories and teeth. Interrater reliability was assessed by repeat measurement on 10% of the sample by an independent blinded assessor (E.C.W.) with the interclass correlation coefficient (ICC) calculated for both incisors and molars. FE‐derived safety thresholds categorized foods as “safe” or “unsafe” and the percentage of safe foods per IDDSI category was calculated.

## 3 Results

### 3.1 Interrater Reliability

Interrater reliability was good to excellent with an ICC of 0.888 for incisors and 0.978 for molars [22].

### 3.2 Bite Force Range, Mean, and Variance

The force required to compress 79 food items and the mean difference between incisor and molar teeth for each category is summarized in Table 2. The lowest bite force for incisors was 0.7 N and for molars was 1.1 N, both when biting crunchy peanut butter (IDDSI 5). The highest bite force for incisors was 56.8 N when biting the cheese stringer (IDDSI 7b), and for molars it was 106.1 N when biting capsicum (IDDSI 7b). The maximum bite force rose from 14.0 N for IDDSI 5 to 56.8 N for IDDSI 7b (388%) for incisors and from 17.8 to 106.1 N (497%) for the molars. The highest molar bite force was 42% higher than the maximum incisor bite force.

**TABLE 2:** Mean bite force values (*N*) for IDDSI Level 5 foods using incisor and molar teeth.

Food item	Mean incisor force (N)	Mean molar force (N)
*IDDSI 5: minced and moist (mean difference between molar and incisor force = 3.0 N, p = 0.049)*		
Crunchy peanut butter	0.7	1.1
Hummus	1.3	1.3
Weet‐Bix (soggy)	2.6	1.7
Mashed potato and gravy	1.4	1.7
Porridge	2.1	2.6
Mashed apple	2.4	3.2
Soften oatmeal (milk)	2.5	3.3
Mashed sweet potato	2.6	4.1
Soft polenta	4.5	4.8
Chopped avocado	3.1	6.7
Cottage cheese	6.2	7.9
Mashed beans	4.2	8.7
Mashed peas	4.7	9.4
Scrambled egg	4.0	9.7
Egg salad	9.6	11.4
Mashed lentils	11.3	11.8
Tinned tuna	10.4	13.0
Ground turkey	14.0	16.6
Mashed banana	2.6	16.9
Beef mince	2.8	17.8
Mean (SD)	4.6 (3.72)	7.7 (5.52)
*IDDSI 6: soft and bite‐sized (mean difference between molar and incisor force = 5.8 N, p = 0.158)*		
Couscous	2.7	2.0
Cooked fish	14.6	4.7
Boiled pumpkin	3.2	5.5
Cooked potato (roasted)	3.3	9.8
Mac and cheese	7.4	10.8
Wet rice	4.3	10.9
Noodles	12.3	12.0
Cooked apple	15.8	13.8
Boiled broccoli	4.9	14.7
Donut	4.1	15.1
Pancakes with maple syrup	17.0	18.3
Stewed pears	9.7	19.0
Stewed zucchini	14.7	20.6
Ravioli (stuffed pasta)	20.4	21.9
Pasta	21.3	30.6
Soft meatballs	40.7	36.6
Dried fig	20.4	39.6
Ham	21.8	44.8
Dinner rolls (no crust)	32.9	51.3
Mean (SD)	14.3 (10.48)	20.1 (14.08)
*IDDSI 7a: easy to chew (mean difference between molar and incisor force = 12.7 N, p = 0.042)*		
Rice pudding	0.9	1.2
Soft muffin	3.7	4.8
Chicken nugget	11.6	6.5
Hot chips	8.7	8.2
Mango	3.5	10.2
Pringle	16.5	14.2
Bread	12.8	15.3
Grape	8.5	16.8
Cucumber	4.8	17.9
Blueberry	11.0	22.0
Soft dumplings	18.2	22.2
Omelet	30.4	22.8
Cooked chicken (tender)	15.2	23.3
Weet‐Bix (soft)	8.1	26.5
Strawberry	14.3	41.2
Pork sausage	41.1	46.3
Tomato	8.8	47.0
Cheese slice	22.7	54.7
Pear	39.0	63.7
Cheerios with milk (1 min)	23.1	72.3
Onion (red)	53.6	85.1
Mean (SD)	17.0 (13.80)	29.6 (23.62)
*IDDSI 7b: regular (mean difference between molar and incisor force = 14.4 N, p = 0.042)*		
Croissant	28.5	19.9
Grilled salmon	24.9	21.0
Popcorn	15.7	21.5
Cooked chicken	14.9	24.1
Sakata cracker	20.5	24.8
Biscuit	25.4	25.6
Chocolate (Cadbury)	33.2	28.8
Sushi	21.2	29.9
Pork tenderloin	28.5	33.6
Pineapple	25.3	38.6
Apple	14.2	39.2
Steak	15.0	44.6
Gummy bear	47.4	50.9
Beef sausage	55.8	57.2
Toast	45.0	57.4
Cheese stringers	56.8	63.8
Pretzel (hard)	34.7	70.6
Cobb salad	42.6	104.9
Capsicum	39.1	106.1
Mean (SD)	31.0 (13.58)	45.4 (26.18)

*Note:* Mean difference between molar and incisor force = 3.0 N, *p* = 0.049.

Abbreviation: IDDSI = International Dysphagia Diet Standardization Initiative.

**FIGURE 4:**
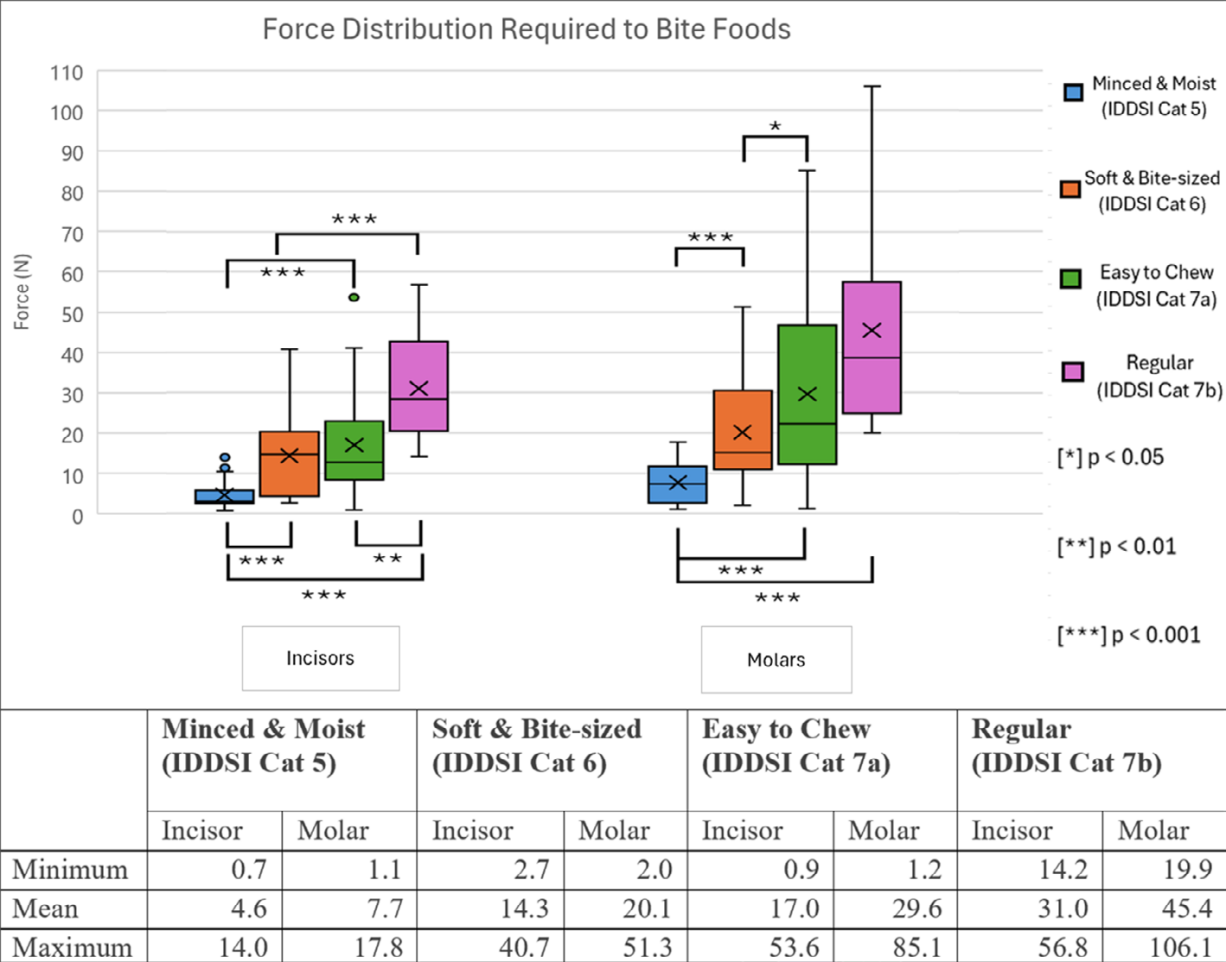
Box plots displaying bite force distribution for each IDDSI Levels (5–7b), separated by incisor and molar loading. Includes an inset table of descriptive statistics for each category. [Color figure can be viewed at wileyonlinelibrary.com]

Mean bite force increased with each food category from 4.6 and 7.7 N for IDDSI 5 to 31.0 and 45.4 N for IDDSI 7b, for incisors and molars, respectively (Figure 4). The mean difference in bite force required for incisors and molars overall was 9.0 N (*p* = 0.004). This was highest for IDDSI 7b (14.4 N, *p* = 0.042) and least for IDDSI 5 (3.0 N, *p* = 0.049) as shown in Figure 4. The mean difference in bite force between IDDSI 5 and IDDSI 7b was 26.3 N for incisors (*p* < 0.001) and 40.7 N for molars (*p* < 0.001). There was a statistically significant difference in the mean bite forces between most IDDSI groups, except for IDDSI 6 and 7a for incisors (mean difference = 9.6 N, *p* = 0.490) and between IDDSI 7a and 7b for molars (mean difference = 15.8 N, *p* = 0.054).

Variance also increased with each food category, with standard deviations (SDs) of 3.72, 13.80, 14.15, and 13.58 for incisors and 5.52, 14.08, 23.62, and 26.18 for molars for IDDSI 5, 6, 7a, and 7b categories, respectively. The greatest relative difference within a category was for IDDSI 5, which ranged from 0.7 N for incisors biting crunchy peanut butter up to 17.9 N for molars biting beef mince, representing a 2457% increase (*p* = 0.017). The greatest absolute difference within a category was for IDDSI 7b, which ranged from 14.2 N for incisors biting apple up to 106.1 N for molars biting capsicum, representing a 647% increase (*p* = 0.015).

### 3.3 FE Analysis

Table 3 shows the peak von Mises stress within the titanium plate when a standard vertical load of 35 N is applied on the incisors (anterior) and molars (posterior). Incisor loading and a greater number of segments resulted in higher stress within the reconstruction plate; however, less stress was observed when there was bone union.

**TABLE 3:** Peak von Mises stress in titanium reconstruction plate and predicted safe biting force from finite element simulations.

	Single‐segment stress (MPa)		Double‐segment stress (MPa)		Triple‐segment stress (MPa)	
	Nonunion	Union	Nonunion	Union	Nonunion	Union
*Peak von Mises stress in the titanium reconstruction plate*						
Anterior loading	190	105	240	110	290	120
Posterior loading	145	90	204	92	220	98
*Predicted safe biting force from FE analysis simulations*						
Anterior loading	50	100	35	95	30	85
Posterior loading	65	115	45	115	40	110

*Note:* “Union” refers to simulated scenarios where bone segments successfully fused; “nonunion” refers to incomplete fusion.

Abbreviations: FE = finite element; MPa = megapascal; N = newton.

Table 3 also outlines the safe loading limits for each reconstruction scenario based on the FE simulations. Single‐segment reconstructions offer the highest safe bite limits, while triple‐segment reconstructions have the lowest, especially under anterior loading. Bone union substantially increased the safe loading limits, allowing 200% (incisors) and 177% (molars) more force for single‐segment reconstructions, 271% and 256% for double segments, and 283% and 275% for triple segments.

### 3.4 IDDSI Category Safety Limits

**FIGURE 5:**
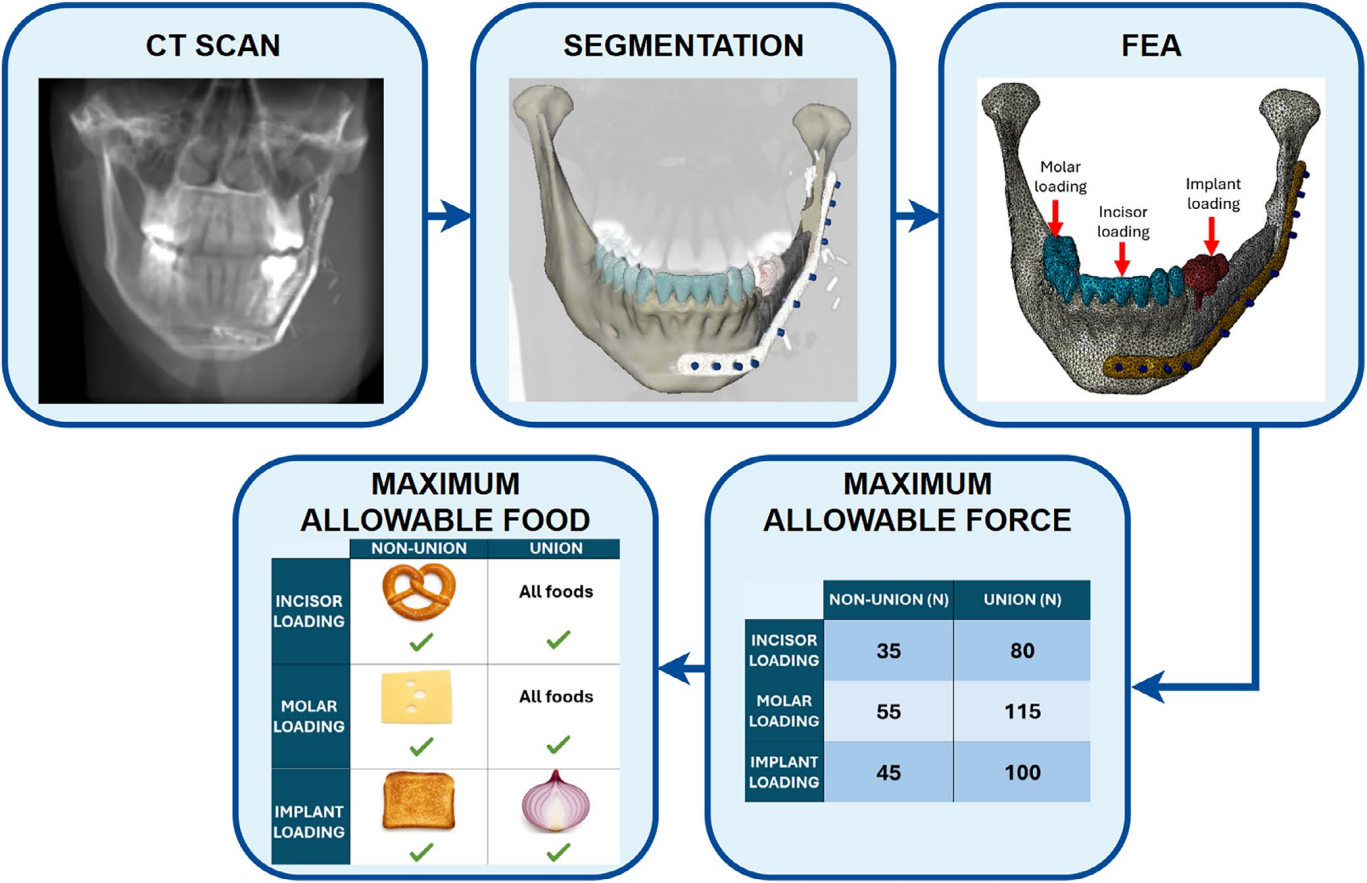
Bar graphs show percentage of foods within each IDDSI Levels (5–7) that fall within safe bite force limits for incisor (anterior) and molar (posterior) loading. Stratified by reconstruction complexity and presence of union. [Color figure can be viewed at wileyonlinelibrary.com]

Figure 5t shows the proportion of foods within each IDDSI category that are predicted to be safe in the six reconstruction scenarios (singe, double, and triple segment with/without bone union) based on FE simulations described above.

For IDDSI 5 foods (minced and moist), 100% were safe regardless of the reconstruction type, union, or location of loading (incisal or molar).

For IDDSI 6 foods (soft and bite sized), 100% were safe with incisal loading for all reconstruction types with union and all single‐segment reconstructions regardless of union. However, in double and triple segment reconstructions, only 95% and 89% were safe without bone union, respectively. For molar loading, all reconstruction types were safe with union. However, without union, only single‐segment mastication was 100% safe, while double and triple segment mastication were safe in 95% and 89% of cases, respectively. Unsafe IDDSI 6 foods included soft meatballs, dinner rolls, and ham.

For IDDSI 7a foods (easy to chew), 100% were safe with incisor loading for all reconstruction types with union. However, only 95% (single), 86% (double), and 81% (triple) were safe without union. For molar loading, 100% of foods were safe across all reconstruction types with bone union. For reconstructions without union, only 90% (single), 71% (double), and 67% (triple) were safe for molar loading. Unsafe IDDSI 7a foods included omelet, pork sausage, pear, Cheerios, tomato, red onion, sliced cheese, and strawberries.

For IDDSI 7b foods (regular), 100% were safe with incisor loading for all reconstruction types with union. However, only 89% (single), 68% (double), and 58% (triple) were safe without union. For molar loading, 100% of foods were safe across all reconstruction types with union, whereas only 84% (single), 63% (double), and 58% (triple) were safe without union. Unsafe IDDSI 7b foods included capsicum, cobb salad, gummy bears, cheese stringers, steak, toast, hard pretzels, beef sausage, and chocolate.

## 4 Discussion

This study is the first to estimate the bite forces needed for foods grouped using the validated IDDSI food texture classification system, providing evidence‐based guidance for selecting safe food textures following mandibular reconstruction in the early (nonunion) and late (union) postoperative settings. This may allow some patients to advance their diet earlier in the postoperative phase and alert clinicians to food types that may never be safe in certain complex reconstruction scenarios. Safe bite force limits vary by factors including the location of the load (incisor or molar), the complexity of the reconstruction, and the presence of bone union. However, there is considerable variation in the force required to masticate different foods both between and within each IDDSI category. We found that in the immediate postoperative period, 57% of regular foods (IDDSI 7b) and 62% of IDDSI 7a foods are safe, which may allow patients to progress toward a more‐normal diet earlier with careful selection. Conversely, following bone union, all IDDSI Categories 5–7b foods proved safe for incisal and molar loading.

Validating the FE simulations performed in this study is challenging considering patient specific variables such as bone density, prior radiotherapy, and the infinite number of reconstruction permutations (e.g., transplanted bone type, location, and length, and plate characteristics and number of screws). However, this complexity makes in vivo and physical modeling impracticable. In contrast, FE parameters can be readily modified, making numerical simulations a powerful tool for biomechanical analysis. Despite this, multiple assumptions were made to reduce the computational demands of the FE analysis. These include fixing the condyles, applying certain bonded and frictional conditions, and loads that were applied to specific teeth, rather than by the muscles of mastication. While based on prior literature [12, 13, 14, 15], these highlight our limited knowledge of the biomechanics of mandibular reconstruction, largely surrounding what muscles remain functional, the rate and efficiency of postoperative chewing, how to quantify different degrees of bone union, and the friction between different segments of bone and hardware. Furthermore, the testing apparatus cannot fully replicate the complexity of typical molar mastication, potentially overestimating the difficulty of chewing certain foods.

Given these limitations, conservative estimates of hardware failure limits were used; FE analysis revealed safe force thresholds of 50 N for incisor (anterior) and 65 N for molar (posterior) loading without union, increasing to 100 N and 115 N with union. These thresholds are based on the risk of titanium plate deformation, which has ample literature to support these modes of failure [18, 19]. Although it is known that bone remodels in response to load [6], there is no consensus on optimal stress levels. Using stronger hardware (thicker plates) may increase safety limits but risks stress shielding and inhibits bone union, the key predictor of long‐term biomechanical integrity. It may also place higher stress on the bone around screws, leading to osteolysis and screw loosening. While this study supports the long‐held assumption that biting into solid foods in the early postoperative phase may be unsafe, FE analysis could be used to develop personalized exercise regimens that actually promote bone union after mandibular reconstruction.

There were significant differences in the force required to bite the different IDDSI categories. IDDSI 5 foods required the least force, averaging 6.2 N, confirming that softer food categories are safe options early in the recovery process. Although IDDSI 5 foods generally had the lowest force, outliers like beef mince exceeded many IDDSI 6 foods, highlighting the need to consider individual food characteristics. Despite this, 100% of the IDDSI 5 foods tested fell under the stress limits regardless of the number of bone segments and presence of union. Also, most IDDSI 6 and IDDSI 7a foods also fell within safe limits; however, individual foods such as soft meatballs and red onion exceeded the safe range. In contrast, IDDSI 7b foods, which contain denser foods and represent a regular diet, required a significantly higher bite force, averaging 38.6 N. The FE analysis demonstrated that 25% of tested foods exceeded the simulated safe limits in complex (two to three segments) reconstructions; however, safety improved with single‐segment reconstructions. Although molars require higher masticatory forces than incisors due to their larger surface area and dull profile, the FE simulations demonstrated higher safe force limits for posterior loading. Thus, determining the safest location for mastication (i.e., incisor or molar) is not always intuitive. Despite this, the molars consistently saw more foods exceed safe limits than incisors for each IDDSI category.

**FIGURE 6:**
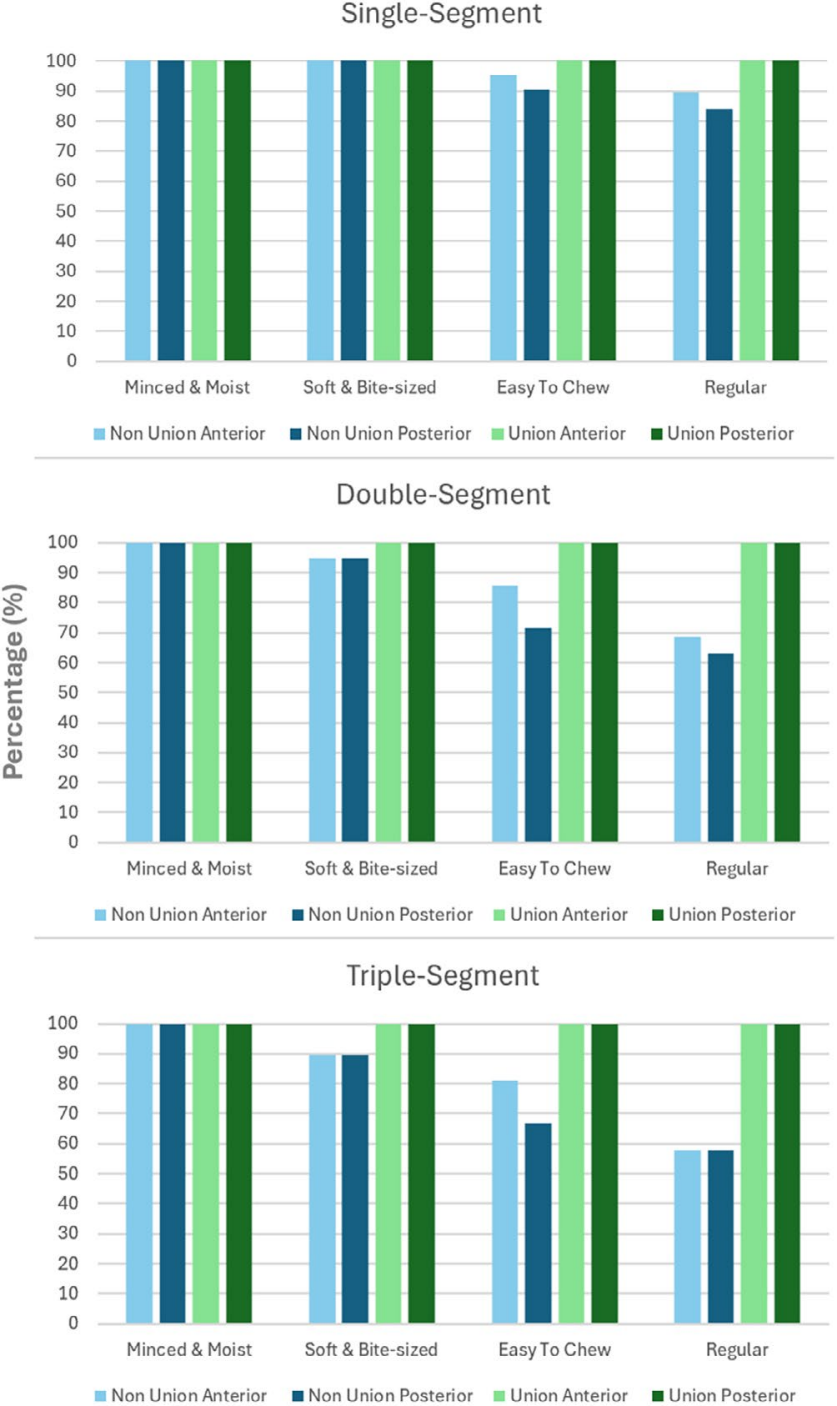
Patient‐based example of the pipeline for developing patient‐specific dietary recommendations postmandibular reconstruction. The figure includes a postoperative CT scan, segmented model, FE analysis results of maximum allowable bite force, and the corresponding maximum allowable foods. [Color figure can be viewed at wileyonlinelibrary.com]

To demonstrate how this approach can be applied in practice, we present an illustrative example of a pipeline for acquiring patient‐specific food recommendations using real patient data (Figure 6). Using postoperative CT data, a segmented mandibular reconstruction model was generated, and FEA was used to establish postoperative safe force thresholds for molar, incisor, and implant loading. These thresholds were then compared against the measured bite force requirements of tested foods outlined in Table 2, which identified the maximum allowable food for the given scenarios of nonunion in the early postoperative period and union in the late postoperative period. In this example, the hardest consistency food that was safe in the early postoperative period was sliced cheese for molar biting, pretzels for incisor biting, and toast for implant biting, suggesting that foods beyond a pureed diet may be appropriate. When union was present, all tested foods were safe for both the molars and the incisors, and red onion was the hardest consistency food safe for the dental implants. This illustrative case highlights the potential for integrating FE modeling into personalized clinical decision‐making, allowing clinicians to prescribe diets tailored to the biomechanical limits of individual reconstructions.

## 5 Conclusions

This foundational data challenges the suitability of the IDDSI dietary categories for mandibular reconstructive patients, particularly those with complex bony reconstructions who are more likely to have coexisting dysphagia. This suggests a need for a bespoke classification system based on bite force and safe‐swallowing texture modifications. Given the nature of mandibular reconstruction, patient‐specific force thresholds should ideally be calculated using image‐based FE analysis. Automating this process could improve clinical applicability. Future studies could investigate optimal mechanical stimulation to promote bone union and rehabilitation, as well as the healing window to develop dynamic and restorative food recommendations.

## Conflicts of Interest

E.K.C., K.C., and J.R.C. hold a patent for Restorabite (Clark J.R., Charters E., Cheng K. “Restorabite.” Australian Provisional Patent PCT/AU2022/050474).

## Data Availability

The data that support the findings of this study are available from the corresponding author upon reasonable request.
